# Probing Temperature Changes Using Nonradiative Processes in Hyperbolic Meta-Antennas

**DOI:** 10.1021/acsaom.4c00098

**Published:** 2024-05-15

**Authors:** Nils Henriksson, Alessio Gabbani, Gaia Petrucci, Denis Garoli, Francesco Pineider, Nicolò Maccaferri

**Affiliations:** † Department of Physics, Umeå University, Linnaeus väg 24, 901 87 Umeå, Sweden; ‡ Department of Chemistry and Industrial Chemistry, University of Pisa, via Moruzzi 13, 56124 Pisa, Italy; § Department of Physics and Astronomy, University of Florence, via Sansone 1, 50019 Sesto, Fiorentino, Italy; ∥ Istituto Italiano di Tecnologia, Via Morego 30, 16163 Genova, Italy; ⊥ Dipartimento di Scienze e Metodi dell’Ingegneria, University of Modena and Reggio-Emilia, Via Amendola 2, 42122 Reggio, Emilia, Italy; # Umeå Centre for Microbial Research, 901 87 Umeå, Sweden; ● Integrated Science Lab, 901 87 Umeå, Sweden

**Keywords:** Hyperbolic meta-antennas, absorption, scattering, temperature sensor, thermoplasmonics, metamaterials

## Abstract

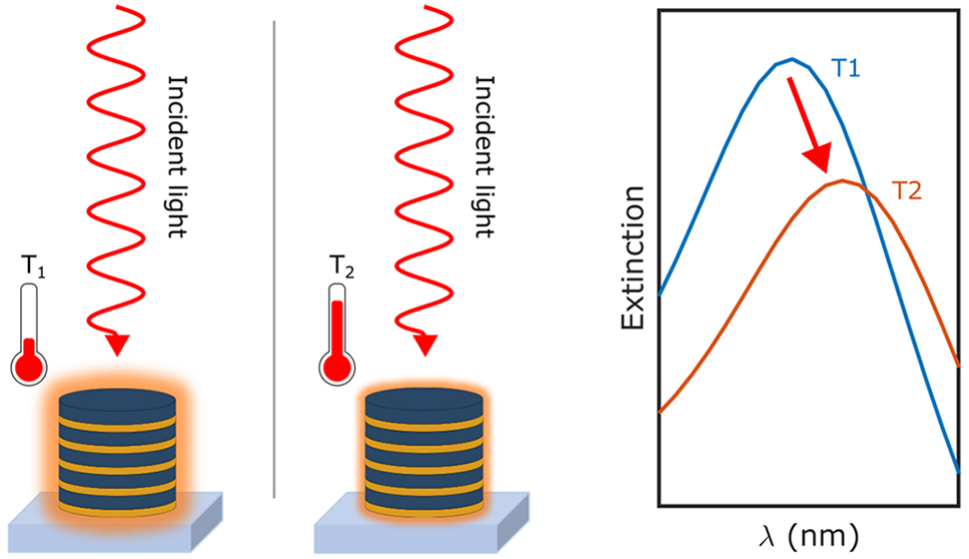

Multilayered metal-dielectric nanostructures display both a strong plasmonic behavior and hyperbolic optical dispersion. The latter is responsible for the appearance of two separated radiative and nonradiative channels in the extinction spectrum of these structures. This unique property can open plenty of opportunities toward the development of multifunctional systems that simultaneously can behave as optimal scatterers and absorbers at different wavelengths, an important feature to achieve multiscale control of light–matter interactions in different spectral regions for different types of applications, such as optical computing or detection of thermal radiation. Nevertheless, the temperature dependence of the optical properties of these multilayered systems has never been investigated. In this work, we study how radiative and nonradiative processes in hyperbolic meta-antennas can probe temperature changes of the surrounding medium. We show that, while radiative processes are essentially not affected by a change in the external temperature, the nonradiative ones are strongly affected by a temperature variation. By combining experiments and temperature-dependent effective medium theory, we find that this behavior is connected to enhanced damping effects due to electron–phonon scattering. Contrary to standard plasmonic systems, a red-shift of the nonradiative mode occurs for small variations of the environment temperature. Our study shows that, to probe temperature changes, it is essential to exploit nonradiative processes in systems supporting plasmonic excitations, which can be used as very sensitive thermometers via linear absorption spectroscopy.

## Introduction

Multilayered metal–insulator structures represent an interesting platform for engineering both the spatial and temporal properties of the electric permittivity in photonic devices.^1^ These structures offer the possibility to bring the refractive index close to zero,^2−4^ enabling novel optical phenomena such as perfect transmission through distorted waveguides,^5^ cloaking^6,7^ and inhibited diffraction.^8^ They also present an almost infinite density of states^9^ and are widely used for nanoscale light confinement and guiding,^10−14^ as well as manipulating scattering, absorption and nonreciprocal propagation of light,^15−22^ generating optical vortex beams^23^ and tailoring optical nonlinearities,^24−30^ as well as for highly sensitive detection^31−35^ and ultrafast all-optical switching.^36−38^ In addition, metal–insulator multilayers display hyperbolic optical dispersion,^39,40^ and thanks to this property they have successfully been implemented as negative index materials^41−43^ and superabsorbers driving resonant gain singularities,^44−47^ as well as for hot-electron generation and manipulation,^48,49^ super resolution imaging,^50^ ultracompact optical quantum circuits,^51^ and lasing.^52^

In this context, it has been shown that multilayered metal-dielectric antennas displaying hyperbolic dispersion have two separated radiative and nonradiative channels^19^ and that this property can be exploited to manipulate electron dynamics on ultrafast time scales^49^ as well as for practical applications such as localized hyperthermia^53^ and enhanced spectroscopy.^21^ The unique property of having two separated spectral regions where either a radiative or a nonradiative process is dominating on the other, and vice versa, can open plenty of opportunities in developing multifunctional systems which can behave at the same time as optimal scatterers and absorbers. This is an essential property to achieve multiscale control of light–matter interactions in different spectral regions for different types of applications, such as optical computing or detection of thermal radiation. Nevertheless, the temperature dependence of the optical properties of these multilayered systems has never been investigated. In this work, we study how radiative and nonradiative channels in hyperbolic meta-antennas can probe temperature changes of the embedding medium. We experimentally show that, while radiative modes are essentially not affected by a change in the external temperature, the nonradiative channel is strongly affected by a temperature variation, displaying a reduction of the absorption cross section together with a broadening/red-shift of the resonance bandwidth/peak. By combining effective medium theory and a temperature-dependent Drude–Lorentz model, we show that this behavior is mainly connected to the damping inside the meta-antennas due to electron–phonon scattering, followed by a red-shift of the plasma frequency of the metallic contribution to the permittivity of the metal-dielectric multilayered systems. Our findings also show that, contrary to standard plasmonic systems, a red-shift of the nonradiative mode occurs for relatively small (of the order of few degrees) variations of the environment temperature. Thus, our study sheds new light on the physics of how radiative and nonradiative channels can probe temperature changes and shows that hyperbolic structures can be used as very sensitive thermometers if we track the spectral variation of their nonradiative processes via linear absorption spectroscopy.

## Results and Discussion

A surface plasmon polariton (SPP) is a light-driven collective oscillation of free electrons localized at the interface between materials with dielectric (ε > 0) and metallic (ε < 0) dispersions. If the interface is flat, as in a thin layer, SPPs can propagate along the interface. When multiple metal/dielectric interfaces supporting SPPs occur within subwavelength separation, the associated coupled electromagnetic field exhibits a collective behavior, which can be modeled by an effective medium approximation (EMA) and the dispersion relation presents a unique anisotropic optical dispersion. More precisely, an effective permittivity tensor ε̂ can be derived such as
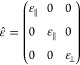
with ε_⊥_ (ε_∥_) the perpendicular (parallel) component with respect to the metal/dielectric interfaces, satisfying ε_⊥_ ε_∥_ < 0 and thus presenting a iso-frequency surface with a hyperbolic shape.^39^

**Figure 1:**
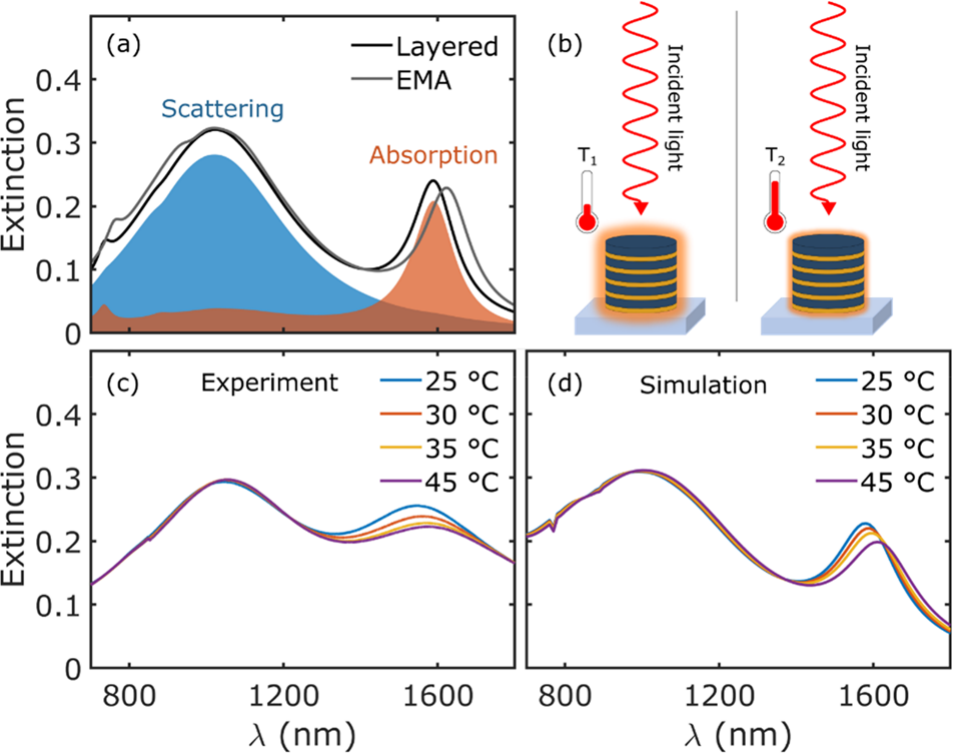
(a) Calculated extinction cross section for a hyperbolic meta-antenna of diameter 250 nm and 5x Au (10 nm)/TiO_2_ (20 nm) layers thickness. The orange and blue areas are the absorption and scattering contributions to the total extinction (black line). The extinction of the structure calculated using the EMA is also plotted (gray curve). (b) Schematic of the experiment showing the decreased absorption (glowing red area surrounding the structure) at increased surrounding temperature. Experimental (c) as well as simulated (d) extinctions at different temperatures. Extinction spectra at four different temperatures have been selected to highlight the main change in extinction as the surrounding temperature increases.

In our study, we investigate the linear optical response of randomly distributed hyperbolic metamaterial (HMM) antennas, known also as hyperbolic meta-antennas. Thus, the average optical response of the system can be associated with the optical response of a single antenna. The optical extinction, which accounts for both scattering (radiative) and absorption (nonradiative) processes, can be described by the EMA (Figure 1a), at different temperatures. The structures are disk-shaped cylinders made of five layers of Au and TiO_2_ with thicknesses of 10 and 20 nm, respectively, and a nominal radius of 125 nm. When illuminated by light, metallic nano-objects exhibit localized plasmon resonances (LSPRs), collective oscillations of the free electron cloud driven by the electromagnetic field of the incident light, in specific spectral regions (Figure 1a). The light extinction in the structures is then greatly enhanced at the wavelengths corresponding to the LSPRs. It has been shown that hyperbolic meta-antennas exhibit a more complex plasmonic response compared to Au antennas with the same geometrical shape and size, featuring two spectrally separated scattering and absorbing modes^19^ (see the contribution of scattering and absorption to the extinction in Figure 1a). However, when the ambient temperature surrounding the structure increases, the refractive index of the meta-antenna changes and thus also the way these structures can interact with light, in particular how they absorb radiation (Figure 1b). The main reason for this change is connected to the fact that the metal building block of our meta-antennas is sensitive to temperature changes, and this sensitivity is amplified by the fact that light–matter interactions at the nonradiative mode are greatly increased.^19,49,53^ Moreover, the size of the antennas increases due to thermal expansion, which in turn lowers the electron density, and thus the plasma frequency,^54^ causing a change in the Au permittivity. With rising temperature, a decrease of the effective mass has been shown to counteract the effect of the lower electron density on the plasma frequency of thin Au films,^54^ causing the plasma frequency to increase. However, we see that our system experiences a clear decrease in the plasma frequency. To investigate the temperature dependence of the optical response of hyperbolic meta-antennas, we performed linear absorption spectroscopy measurements to characterize the extinction as a function of wavelength at different ambient temperatures (see Methods and Supporting Information for details about the experiments and the experimental setup, respectively).

As illustrated in Figure 1c, the extinction of our meta-antennas decreases at the absorption mode (thus where nonradiative processes dominate) with increasing temperature. At the same time, the extinction at the scattering mode remains almost unchanged for the temperature variation range we are considering here, that is, from room temperature (25 °C) up to 45 °C. In addition to a decrease of extinction at the absorption mode, we can also appreciate a broadening and a red-shift of the resonance. The first effect can be explained by considering an increased damping of the electrons inside the metal, while the red-shift needs a more detailed analysis.

**Figure 2:**
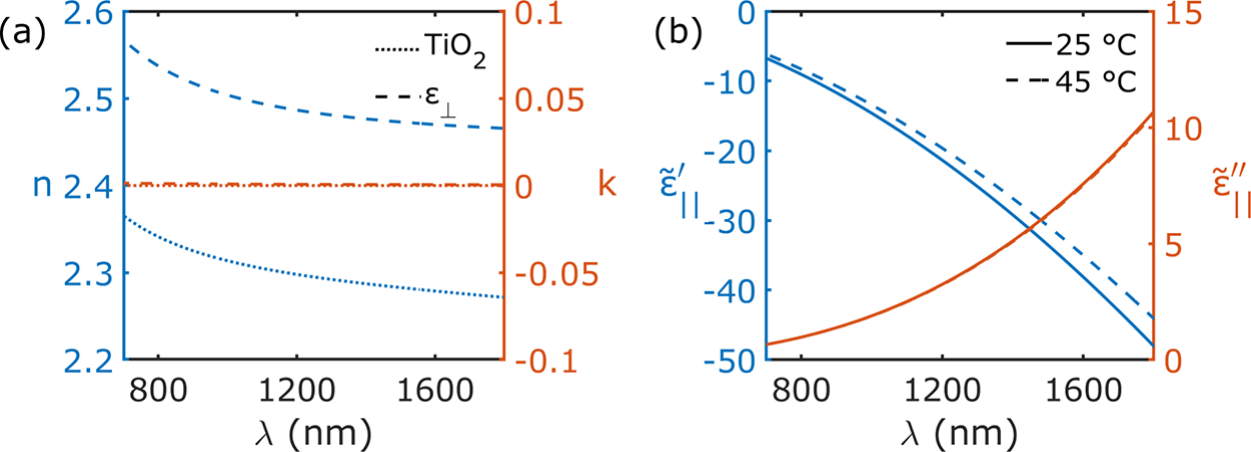
(a) The experimental refractive index of TiO_2_^64^ (dotted curve) and the refractive index calculated using ε_⊥_ (dashed curve) assuming a constant value of 2 for the TiO_2_ refractive index. (b) The temperature-dependent complex permittivity ε̃_∥_ at 25 and 45 °C.

To understand the physics underlying the temperature-dependence behavior of both nonradiative and radiative processes in hyperbolic meta-antennas, we performed finite element method (FEM) simulations using the commercial software COMSOL Multiphysics, utilizing the wave optics module to calculate the steady state extinction as a function of the incident light wavelength (see Figure 1d). The system was modeled using the EMA, with the permittivity components calculated as^22,55^
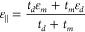
1
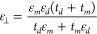
2where ε_*m*,(*d*)_ is the Au (TiO_2_) permittivity, and *t*_*m*,(*d*)_ is the layer thickness of Au (TiO_2_). In our study, we focus on wavelengths where intraband transitions are the main contribution to the permittivity;^22^ thus, we used a Drude model to describe Au permittivity.^56^ In Figure S3, we plot the in-plane component of the hyperbolic permittivity, ε_∥_ as defined in eq 1, thus with no temperature dependence. As it can be inferred by looking at the figure, the real part (blue curves) is negative, thus indicating that in the plane the hyperbolic meta-antennas have a metallic behavior. It is worth noting that the refractive index of TiO_2_ is not constant in the wavelength range considered (see blue curve in Figure 2a).^57^ However, instead of optimizing the size and shape of our modeled structure to match the location of the experimental extinction peaks, we slightly altered the refractive index of the dielectric. This does not change the physics underlying the observed phenomena. For the dielectric material in our EMA model, we used a constant refractive index of 2. In Figure 2a, we plot both the experimental refractive index of TiO_2_ from ref (57) and the one calculated using the out-of-plane component ε_⊥_ of the EMA with the assumed constant value for the TiO_2_ refractive index. The two quantities behave similarly, and the imaginary part of the refractive index, the loss coefficient, is almost zero over the whole spectral range. This indicates that our out-of-plane refractive index is that of an insulator, and the variation of TiO_2_ refractive index with temperature is known to be very negligible in the range of temperatures we are considering here.^58−61^

Thus, we can disregard contributions to the optical extinction due to the temperature dependence of the out-of-plane component of our hyperbolic permittivity and consider only the contributions from the in-plane (metallic) component ε̃_∥_.
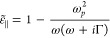
3We made a fit of eq 3 to the in-plane component ε_∥_ in eq 1, which is based on a Drude model of the Au permittivity.^56^ It is worth mentioning here that, since we are in the NIR region, it is reasonable to assume that only the 6sp-band electrons of Au are affected by a temperature change, and thus, they can be treated with a free electron model. For this reason, we can also infer that the main change will be on the damping rather than on the interband contribution from the 5d band, as that contribution needs a large amount of energy to be “perturbed” and thus to contribute to a change in the permittivity of the system.

**Figure 3:**
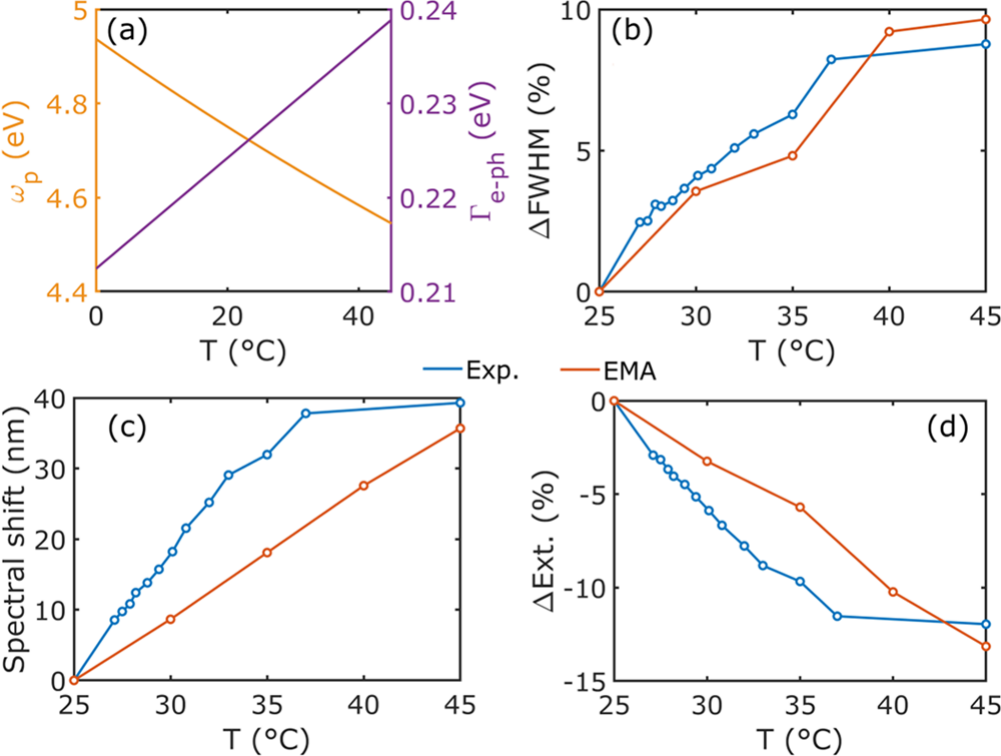
Changes at the absorbing mode driven by the temperature change. (a) Temperature-dependent plasma frequency and damping in eV. Effects of changing the surrounding medium temperature on (b) FWHM, (c) spectral position of the extinction peak, and (d) peak extinction intensity for both the experimental (blue curves) and the simulated (orange curves) cases.

By using the Drude model for the Au permittivity and a constant refractive index for the TiO_2_, we ensured that the Drude fit described in eq 3 was consistent for both the imaginary and the real part. In case a more sophisticated model is used for the Au permittivity, such as the Brendel–Bormann model, a simple Drude fit is inconsistent for the real and the imaginary part. Thus, the values of the parameters in our Drude fit (eq 3) were found to be *ω*_*p*_ = 4.937 eV and Γ = 0.0184 eV. However, when using a simple Drude model for the Au permittivity, the damping is underestimated. To account for this, we increased the damping to Γ = 0.15 eV, which well reproduced the spectra from the initial EMA model in Figure 1a, which in turn is based on the Brendel–Bormann permittivity model on Au.^62^ This approach was used to enable precise modeling of the temperature effects on both the plasma frequency and damping constant. We thus built a temperature-dependent model for ε̃_∥_ following previous works done on metals.^54,63^ Following similar procedures, we derived the temperature dependences of plasma frequency and damping constant as
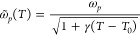
4
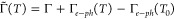
5where *T*_0_ is the room temperature and *ω*_*p*_ the plasma frequency, γ the thermal expansion coefficient, and Γ the damping used in eq 3. The temperature-dependent damping, due to electron–phonon scattering at temperatures much larger than the Debye temperature of Au, *θ*_*D*_ = 170 K, can be calculated as^54^
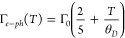
The thermal expansion coefficient γ and the electron–phonon scattering rate Γ_0_ were used as fitting parameters to match the behavior of the experimental results. We found that γ = 4 × 10^–3^ K^–1^ and Γ_0_ = 0.1 eV gave the best fit. Inserting eqs 4 and 5 into eq 3 provided us with a temperature-dependent metallic-like permittivity ε̃_∥_. The temperature-dependent real and imaginary parts of ε̃_∥_ (ω,*T*) are displayed in Figure 2b. We only considered thermal effects on the plasma frequency and the electron–phonon-induced damping as they are the main contributors to the change in permittivity, as previously shown for pure metallic structures,^63^ where both electron–electron and phonon–phonon scattering processes can be ignored. The temperature dependences of both quantities are displayed in Figure 3a. By comparing the experimental results (Figure 1c) with our simulations (Figure 1d), it is clear that our simple model replicates the behavior of the extinction spectrum when the ambient temperature increases; thus, a more complex model that includes the electron–electron, phonon–phonon, and even electron-surface scattering-induced damping is not necessary (more details about the simulations of absorption and scattering cross sections can be found in Methods).

A full evaluation and comparison between the experimental and the numerical results at the absorption mode were performed by computing the relative temperature-induced change of the full width at half-maximum (FWHM) (Figure 3b) and the spectral shift of the resonance peak (Figure 3c), as well as the change of peak extinction (Figure 3d). This was done using a double Lorentzian fit (see the Supporting Information for more details). The figure shows that our model can predict the change in FWHM accurately, while the red-shift as well as the decrease in magnitude show similar changes between 25 and 45 °C, albeit with a more linear behavior than the experimental counterpart. In our model, we assume a constant volume thermal expansion coefficient γ. For Au, this is a good approximation, as the thermal expansion coefficient has a linear correlation to the heat capacity, which above the Debye temperature (170 K) is close to constant. For TiO_2_, however, the Debye temperature is 562 K.^65^ Its heat capacity, and thus thermal expansion coefficient, is therefore not constant, which might induce nonlinear behavior that is not considered in our model.

**Figure 4:**
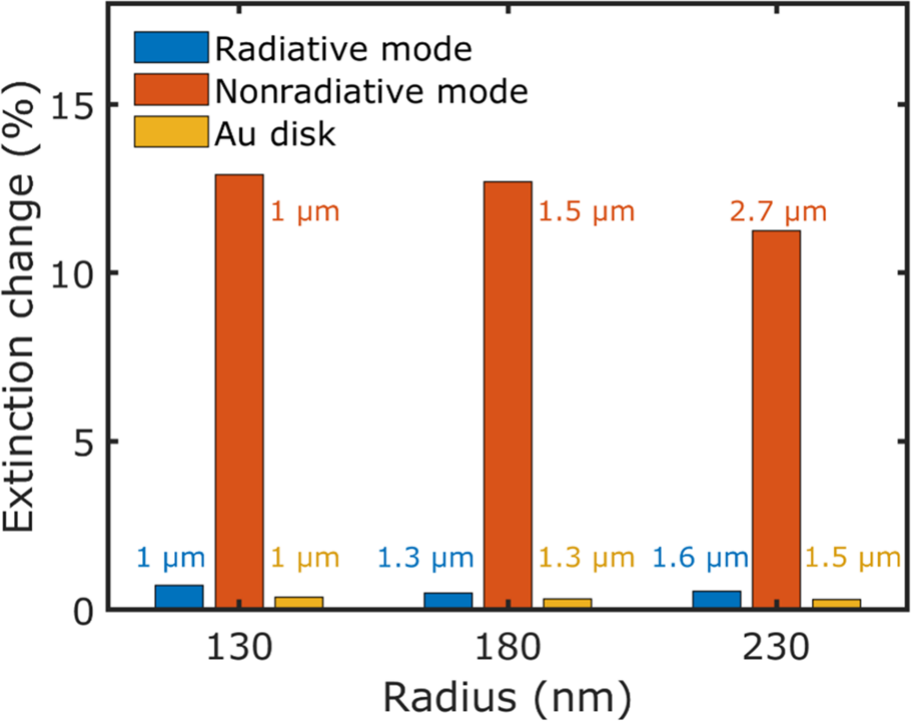
Relative extinction changes between 25 and 45 °C at the scattering and absorbing mode of antennas with different diameters, calculated with the EMA model, as well as the corresponding extinction change of a gold disk of the same radius as the HMM structure with a height of 50 nm. The simulation of the Au disk was performed using the Brendel–Bormann permittivity for Au.^62^ The wavelength of each resonant mode is displayed in the figure.

It is worth mentioning here that, given that the refractive index of TiO_2_^64^ remains almost constant and that Au^62^ permittivity can be described by the Drude model between λ = 1 μm and λ = 5 μm, this implies that out model is valid further into the infrared spectrum. Thus, we simulated hyperbolic meta-antennas where we increased the radius of the structures to red-shift both peaks of the extinction spectrum and investigate whether our system behaves in the same way also when both radiative and nonradiative processes move toward the mid infrared. In Figure 4, we show the extinction change at the scattering and absorption modes between 25 and 45 °C for structures with radii 130, 180, and 230 nm. While the absorption mode is still very sensitive to temperature changes, the scattering mode is still not affected much by a temperature variation around room temperature. Thus, our hyperbolic meta-antennas can be used as both sensitive thermometers of macroscopic temperature changes if nonradiative processes are optically detected, for instance via linear absorption spectroscopy, and for thermal sensing in the infrared spectral region. Finally, we want to highlight that it is not possible to achieve this feature with standard plasmonic antennas made of gold and with the same geometrical parameters such that they resonate in this spectral range (see Figure 4). This is due to the fact that scattering is the dominant extinction pathway in these structures. In Figure S4, the underlying spectra for Figure 4 are displayed.

## Conclusion

In summary, we have experimentally studied how the linear optical response of hyperbolic meta-antennas changes by varying the surrounding medium temperature from room temperature up to 45 °C. We found that the nonradiative contribution (absorption) to the extinction is sensitive to temperature changes, while the radiative contribution (scattering) is not sensitive in the range of temperatures considered. By combining effective medium approximation and temperature-dependent Drude model, we showed that the main effects affecting the linear optical response at the absorption peak are related to a drastic change of the electron damping and the plasma frequency due to electron–phonon scattering processes, and this effect results in a reduction of the absorption cross section together with a broadening/red-shift of the resonance width/peak. Thus, nonradiative processes in hyperbolic meta-antennas, compared to their counterparts in pure metallic plasmonic structures, are very sensitive to temperature changes and can be eventually used as temperature detectors in devices using optical read-out schemes. An intriguing extension of the study is to investigate the temperature dependence of Type-I HMM antennas,^66^ for which a similar approach can be used. Finally, we envision that these types of nanostructured metamaterials can be implemented as thermal radiation sensors in future all-optical technologies.

## Methods

### Fabrication

The multilayer pillars were fabricated following a few steps of process: electron beam lithography was performed on a MMA:PMMA(950 K) bilayer spin coated at 3000 rpm on a glass substrate; after the exposure and successive resist development in IPA:DI Water (2:1), electron-beam evaporation was used to deposit the different layers in the system. The thicknesses of the different layers were first calibrated by performing spectroscopic elipsometry on sample where a single layer was deposited. Finally, the lift-off was performed by immersing the sample in acetone for 30 min.

### Experiments

UV–vis-NIR extinction spectra were acquired using a JASCO V670 commercial spectrophotometer working in transmission configuration. A metal ceramic heater element (HT19R, Thorlabs) was placed in contact with the sample substrate and used to heat the sample during the measurements of the spectra. The ceramic has a 4 mm hole that allows light to pass through the sample. The sample temperature is controlled with a 100 Ohm resistive temperature sensor placed on the ceramics. The temperature was kept constant during each spectrum with maximum variations of 1 °C. Extinction spectra were acquired at temperatures between 25 and 45 °C.

### Numerical Calculations

The steady-state extinction spectra are calculated from FEM simulations in COMSOL Multiphysics on the full 150 nm high disk with a 130 nm radius, utilizing the EMA. Using scattered field formulation in the frequency domain, the absorption and scattering cross sections were calculated as
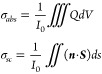
where *I*_0_ is the intensity of the incident light, *Q* is the power absorbed by the structure, and (***n*** · ***S***) is the Poynting vector in the normal direction of the surface of the particles. By dividing both quantities by the cross-sectional area of the computational domain with normal along the direction of the light, we obtained absorption, scattering and therefore extinction values similar to the experimental results. An anisotropic permittivity tensor ε̂ with a temperature-dependent in-plane component ε̃_∥_(*T*) was used to account for the effect of the temperature change on the optical response of the meta-antennas.

## Data Availability

The authors declare that the data supporting the findings of this study are available within the paper, and its Supporting Information. The raw data are available from the corresponding author upon request.

## Supporting Information Available

Details about FWHM calculations and EMA simulation using temperature-independent gold permittivity. Figures show experimental setup, example of Lorentzian fit, parallel component of temperature-independent EMA, and extinction spectrum for structures of different sizes (PDF)
