# Supplementary material for: Probing Temperature Changes Using Nonradiative Processes in Hyperbolic Meta-Antennas
Source: ACS Appl Opt Mater. 2024 May 15;2(12):2469–75. doi: 10.1021/acsaom.4c00098 (PMC11686678; doi:10.1021/acsaom.4c00098)
Supplement: Supplementary file 1 — ot4c00098_si_001.pdf [file ot4c00098_si_001.pdf]

Supporting information

Probing Temperature Changes Using Nonradiative Processes in Hyperbolic Meta-Antennas

Nils Henriksson[1], Alessio Gabbani[2], Gaia Petrucci[2], Denis Garoli[3], Francesco Pineider[2], and Nicolò Maccaferri[1,6]*

[1]Department of Physics, Umeå University, Linnaeus väg 24, 901 87 Umeå, Sweden

[2]Department of Chemistry and Industrial Chemistry, University of Pisa, via Moruzzi 13, 56124, Pisa (Italy)

[3]Department of Physics and Astronomy, University of Florence, via Sansone 1, 50019, Sesto Fiorentino (Italy)

[4]Istituto Italiano di Tecnologia, Via Morego 30, 16163 Genova (Italy)

[5]Dipartimento di Scienze e Metodi dell'Ingegneria, University of Modena and Reggio-Emilia, Via Amendola 2, 42122 Reggio Emilia (Italy)

[6]Umeå Centre for Microbial Research, 901 87 Umeå, Sweden

*nicolo.maccaferri@umu.se

1. Calculating the FWHM

To compare the effect of the temperature in the experimental results (Figure 1c in the main paper) with the simulations in Figure 1d (main paper), we made a double Lorentzian fit on the different data,

$$f(\lambda) = \frac{I_0}{\left[1 + \left(\frac{x - x_0}{\gamma_0}\right)^2\right]} + \frac{I_1}{\left[1 + \left(\frac{x - x_1}{\gamma_1}\right)^2\right]},$$

where $\{I_{0,1}, x_{0,1}, \gamma_{0,1}\}$ are fitting parameters. $I_{0,1}$ are the magnitudes of each peak, $x_{0,1}$ the position of the peaks and $2\gamma_{0,1}$ the full width at half maximum (FWHM) of each peak (see Figure S2).

2. Effective medium approximation

In our work, we presented a new, simple method to numerically estimate the effect of a changing ambient temperature on multilayered nanoantennas. However, as a validation of the Effective Medium Approximation (EMA) approach for this system, we performed a simulation using the EMA with Au permittivity by Rakić et al.[1], and the anisotropic permittivity tensor implemented as described in the main text. We also made a simulation of a layered structure. The results are displayed in Figure 1a, showing a similar extinction spectrum for both cases.

Supporting figures

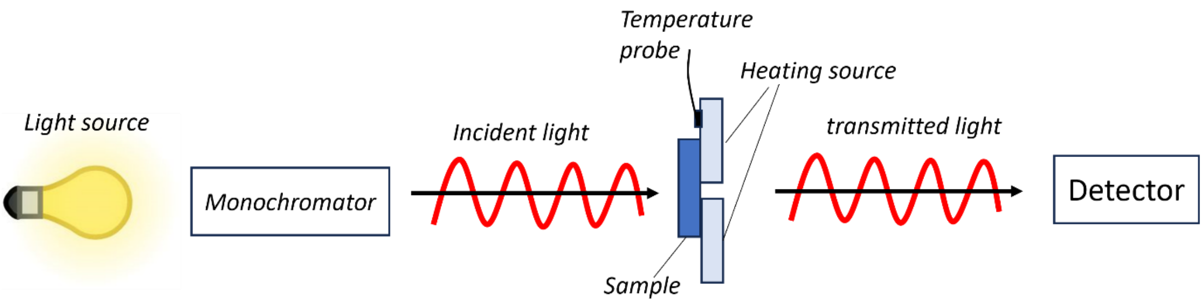

Figure S1. Experimental setup used in the experiments.

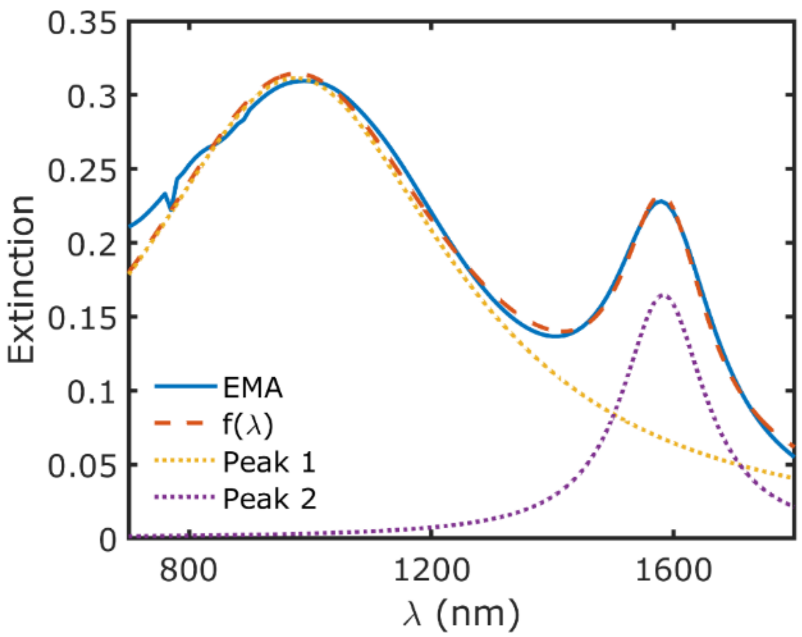

Figure S2. Example of a double Lorentzian fit. The dashed line shows the fit of the data, and the dotted curves show the fit of each peak.

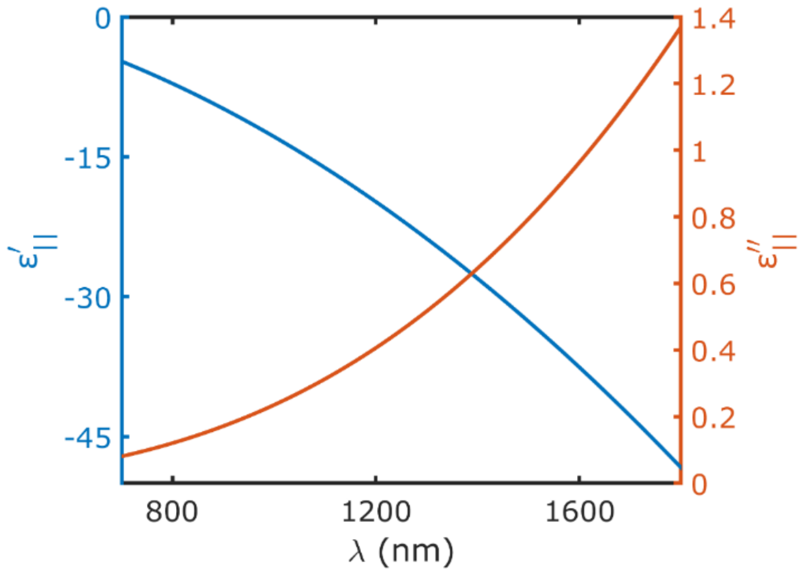

Figure S3. Wavelength dependence of real (blue) and imaginary (orange) part of the parallel component of the EMA of the antennas. The refractive index of TiO_2 was set to 2, while we used a Drude model for the Au permittivity.[2]

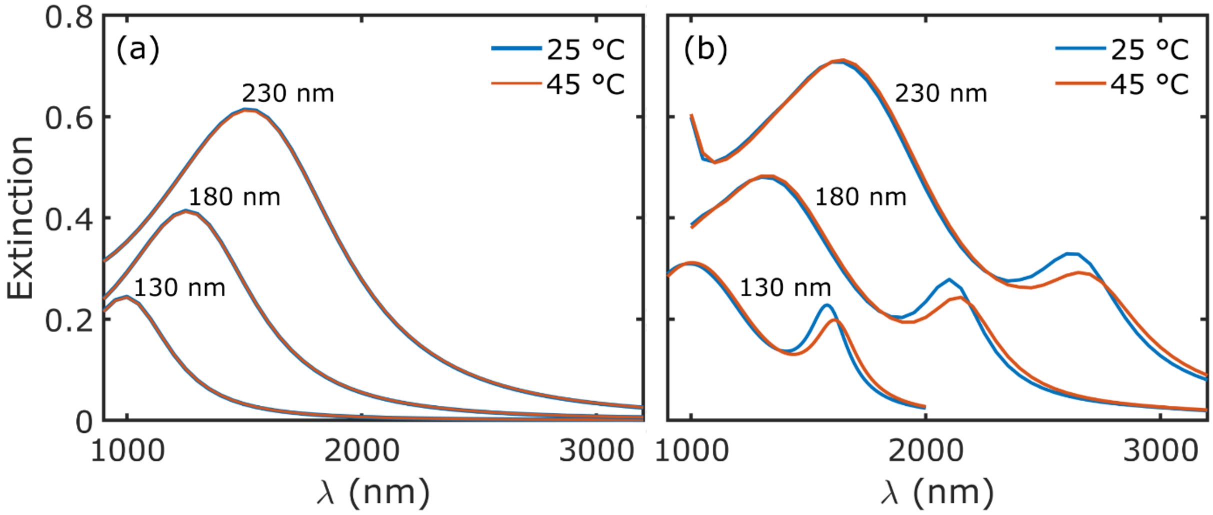

Figure S4. Simulated extinction spectrum of (a) Au cylinders and (b) HMM antennas of different sizes and in different temperatures. For Au, the Brendel-Bormann permittivity model was used,[1] while we used the EMA model described in the main text for the HMM antennas.

References

(1) Rakić, A. D.; Djurišić, A. B.; Elazar, J. M.; Majewski, M. L. Optical Properties of Metallic Films for Vertical-Cavity Optoelectronic Devices. *Appl. Opt., AO* **1998**, *37* (22), 5271–5283. https://doi.org/10.1364/AO.37.005271.

(2) Blaber, M. G.; Arnold, M. D.; Ford, M. J. Search for the Ideal Plasmonic Nanoshell: The Effects of Surface Scattering and Alternatives to Gold and Silver. *J. Phys. Chem. C* **2009**, *113* (8), 3041–3045. https://doi.org/10.1021/jp810808h.
